# T-lymphoblastic Leukemia/lymphoma presenting as pericardial tamponade: a case report and literature review

**DOI:** 10.1093/omcr/omag065

**Published:** 2026-11-02

**Authors:** Laith Rhabneh, Mohammed Alkhanafsa, Ra’ed Ababneh, Shahd Qaddour, Jamil Wafi, Emad Algorani, Tareq Alzughayyar, Hashim Mohammad, Hussam Telfah

**Affiliations:** Department of Internal Medicine, Hackensack Meridian Ocean University Medical Center, Brick, United States; Department of Histopathology, Hamad Medical Corporation, Doha, Qatar; Department of Internal Medicine, Hackensack Meridian Ocean University Medical Center, Brick, United States; Diagnostic Radiology Department, King Abdullah University Hospital, Irbid, Jordan; Department of Histopathology, Hamad Medical Corporation, Doha, Qatar; Department of Histopathology, Hamad Medical Corporation, Doha, Qatar; Department of Histopathology, Hamad Medical Corporation, Doha, Qatar; Department of Histopathology, Hamad Medical Corporation, Doha, Qatar; Department of Histopathology, Hamad Medical Corporation, Doha, Qatar

**Keywords:** cardiovascular disorders, oncology, emergency medicine

## Abstract

Background: T-lymphoblastic leukemia/lymphoma (T-ALL/LBL) is a rare, aggressive hematologic malignancy that typically affects young adults and often presents with a mediastinal mass or bone marrow involvement. Case presentation: A 35-year-old male with no prior medical history presented with progressive shortness of breath, dry cough, fever, night sweats, and weight loss. He was tachycardic and in respiratory distress, with jugular venous distention and muffled heart sounds. Imaging revealed a large anterior mediastinal mass with pleural and pericardial effusions. Echocardiography confirmed tamponade physiology, prompting emergent pericardiocentesis, which drained 200 ml of fluid; cytology suggested a lymphoid malignancy. Subsequent CT, PET/CT, and tissue studies confirmed T-ALL/LBL without bone marrow involvement. He began chemotherapy and was discharged in stable condition. Conclusion: Although rare, T-ALL/LBL should be considered in unexplained pericardial effusions.

**Key Clinical Message**

Pericardial tamponade may be the first manifestation of T-lymphoblastic leukemia/lymphoma. Clinicians should maintain a high index of suspicion for underlying malignancy in unexplained pericardial effusions to enable prompt diagnosis and life-saving intervention. Early multidisciplinary management is crucial to improve outcomes in such rare and aggressive presentations.

## Introduction

T-lymphoblastic leukemia/lymphoma (T-ALL/LBL) is an aggressive hematologic malignancy arising from precursor T-cells, primarily affecting children and young adults [1]. T-ALL/LBL accounts for about 20% of ALL in adults [2]. T-cell lymphoblastic lymphoma (T-LBL) and T-cell acute lymphoblastic leukemia (T-ALL) are considered the same disease [3]. However, in clinical practice, the distinction between T-cell acute lymphoblastic leukemia (T-ALL) and T-cell lymphoblastic lymphoma (T-LBL) is determined by the extent of bone marrow involvement. T-ALL is diagnosed when bone marrow involvement exceeds 25% blasts, with or without a mediastinal mass, whereas T-LBL is diagnosed when there is a mediastinal or other mass, accompanied by less than 25% blasts in the bone marrow [3].

This hematologic malignancy can present with a wide range of clinical features, the most common of which are leukemic features, hypercalcemia, lymphadenopathy, hepatosplenomegaly, skin lesions and mediastinal masses. In addition, it can initially present as cardiac tamponade [4].

Pericardial tamponade is rare in T-ALL/LBL, though pericardial involvement can occur. While specific incidence data for tamponade in T-ALL/LBL are limited, the available literature suggests this is an uncommon but recognized complication. Pericardial effusions occur in approximately 23% of patients with acute lymphoblastic leukemia who undergo echocardiographic evaluation, though most are minimal in size and asymptomatic [5].

Pericardial effusion and cardiac tamponade in hematologic malignancies often result from direct tumor infiltration, high disease burden, or secondary inflammatory processes. The presence of a large mediastinal mass with pericardial involvement can pose a therapeutic challenge, requiring prompt intervention [6].

In this case report, we are reporting a 35-year-old previously healthy man who initially presented with cardiac tamponade due to T-ALL/LBL. This case report underscores the importance of considering unusual manifestations of T-ALL/LBL and the need for early intervention.

## Case report

A 35-year-old male patient, with no past medical history, presented to the emergency department with progressive shortness of breath over three months. The patient also complained of a persistent dry cough, fever, night sweats, and significant unintentional weight loss. The patient reported no chest pain. The patient reported no current medications or illicit drug use. He is single, lives with friends, and has no family history of malignancy.

On physical examination, the patient appeared uncomfortable and exhibited difficulty breathing. He was afebrile (36.4°C) and his blood pressure was 111/75 mmHg. Pulse rate was 132 beats per minute, respiratory rate was 26 per minute, and oxygen saturation of 91% on room air.

The head and neck examination was notable for jugular venous distention. Cardiac examination revealed muffled heart sounds without additional sounds. Chest examination showed engorged veins and diminished breath sounds on the right side, while abdominal exam was significant for epigastric tenderness, right upper quadrant tenderness, and hepatomegaly, where the liver was palpable 3 cm below the right costal margin.

Further work-up was largely unremarkable, showing a normal complete blood count, as well as preserved renal and liver function. Notable findings included a mildly elevated uric acid level of 463 μmol/l and a lactate dehydrogenase (LDH) of 247 U/l.

Chest X-ray showed a prominent dense right hilar shadow with a large right paratracheal soft tissue mass, suggestive of enlarged lymph nodes. Additionally, moderate right-sided pleural effusion with underlying hilar vascular congestion was noted, along with subtle blunting of the left costophrenic angle. Cardiac size assessment was limited (Fig. 1).

**Figure 1:**
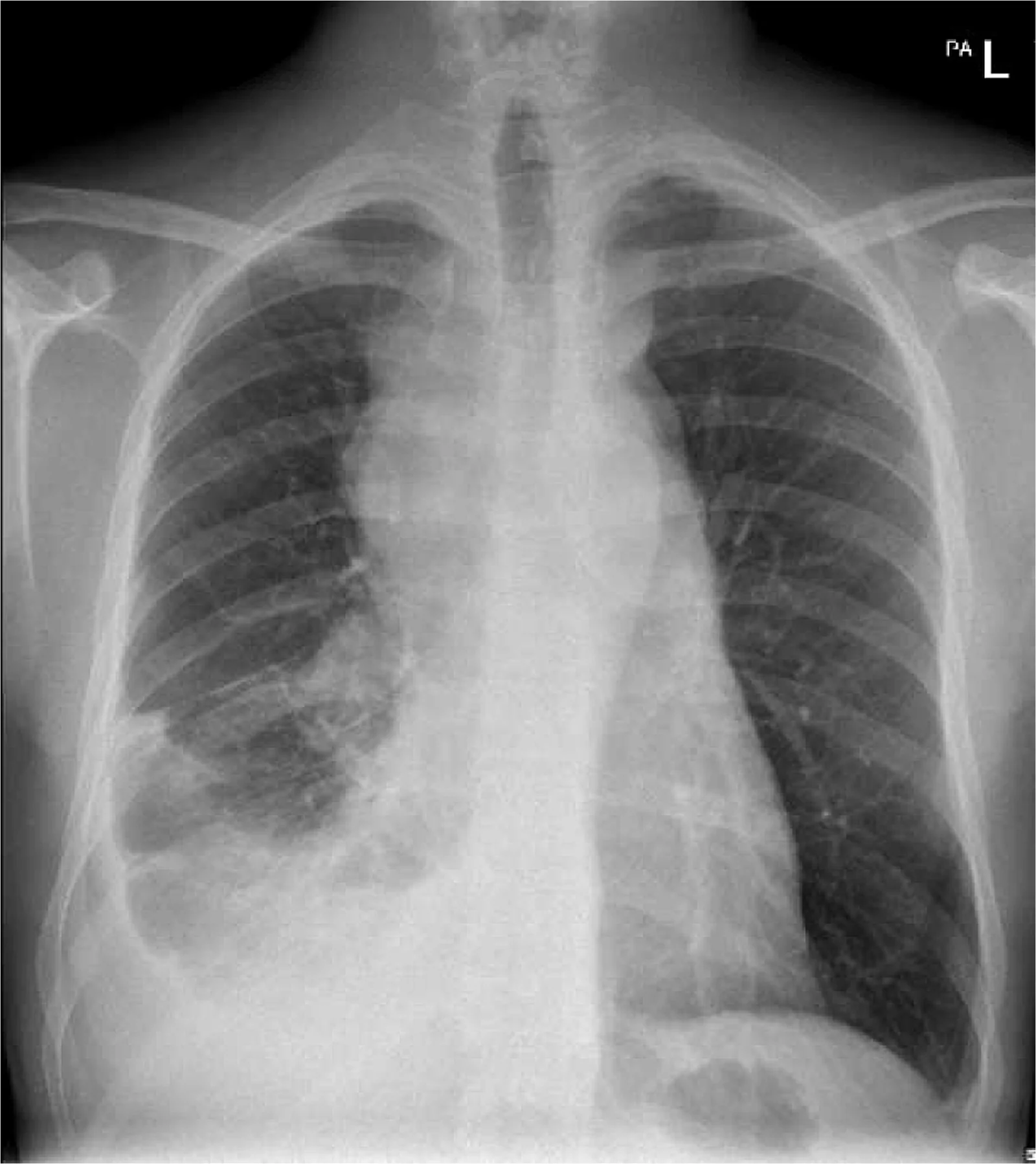
The chest X-ray showing right paratracheal soft tissue mass, the right pleural effusion, and blunting of the left costophrenic sulcus.

ECG findings included sinus tachycardia, low voltage, and electrical alternans. Transthoracic echocardiography confirmed a large circumferential pericardial effusion with features of cardiac tamponade (Fig. 2).

**Figure 2:**
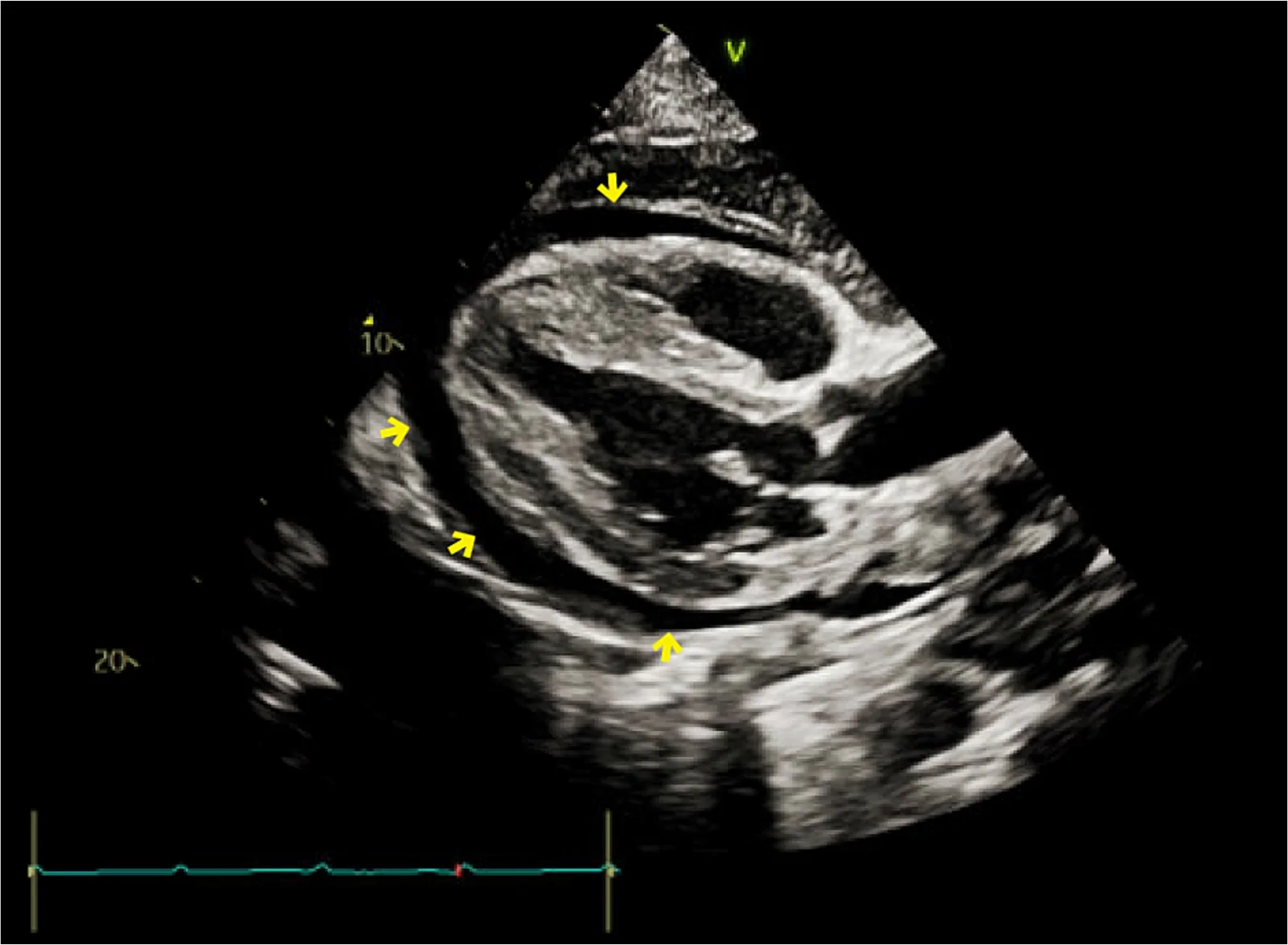
Transthoracic echocardiography showing a large circumferential pericardial effusion.

Emergent pericardiocentesis was performed, draining 200 ml of fluid. Pericardial fluid analysis revealed a hemorrhagic, exudative effusion with a markedly elevated nucleated cell count (13 750 cells/μl), predominantly composed of abnormal lymphoid cells (95%). Biochemical evaluation demonstrated extremely low glucose levels (<0.1 mg/dl) and elevated protein concentration (40.8 g/l), findings consistent with malignant pericardial involvement. Cytologic examination showed highly cellular smears with numerous large, abnormal cells consistent with a lymphoid origin, along with scattered neutrophils and macrophages. Nuclear debris and frequent apoptotic bodies were present, indicating rapid cell turnover. Overall, the findings were highly suggestive of malignant lymphoid involvement of the pericardium.

Following pericardiocentesis and hemodynamic stabilization, transthoracic echocardiography revealed a preserved left ventricular ejection fraction of 65% and normal wall motion. Serum troponin levels were within normal limits. A contrast-enhanced Computed Tomography (CT) scan of the thorax revealed a large loculated anterior mediastinal mass (102 × 94 × 183 mm) with necrotic areas extending from the lower neck, encasing major vessels and causing mild attenuation. It also showed bilateral hilar lymphadenopathy and discrete right paracardial masses, possibly lymph nodes. There is bilateral pleural effusion (mild on the left, moderate on the right), pericardial effusion, and right perihilar atelectatic changes. No fat content or calcifications seen (Fig. 3).

**Figure 3:**
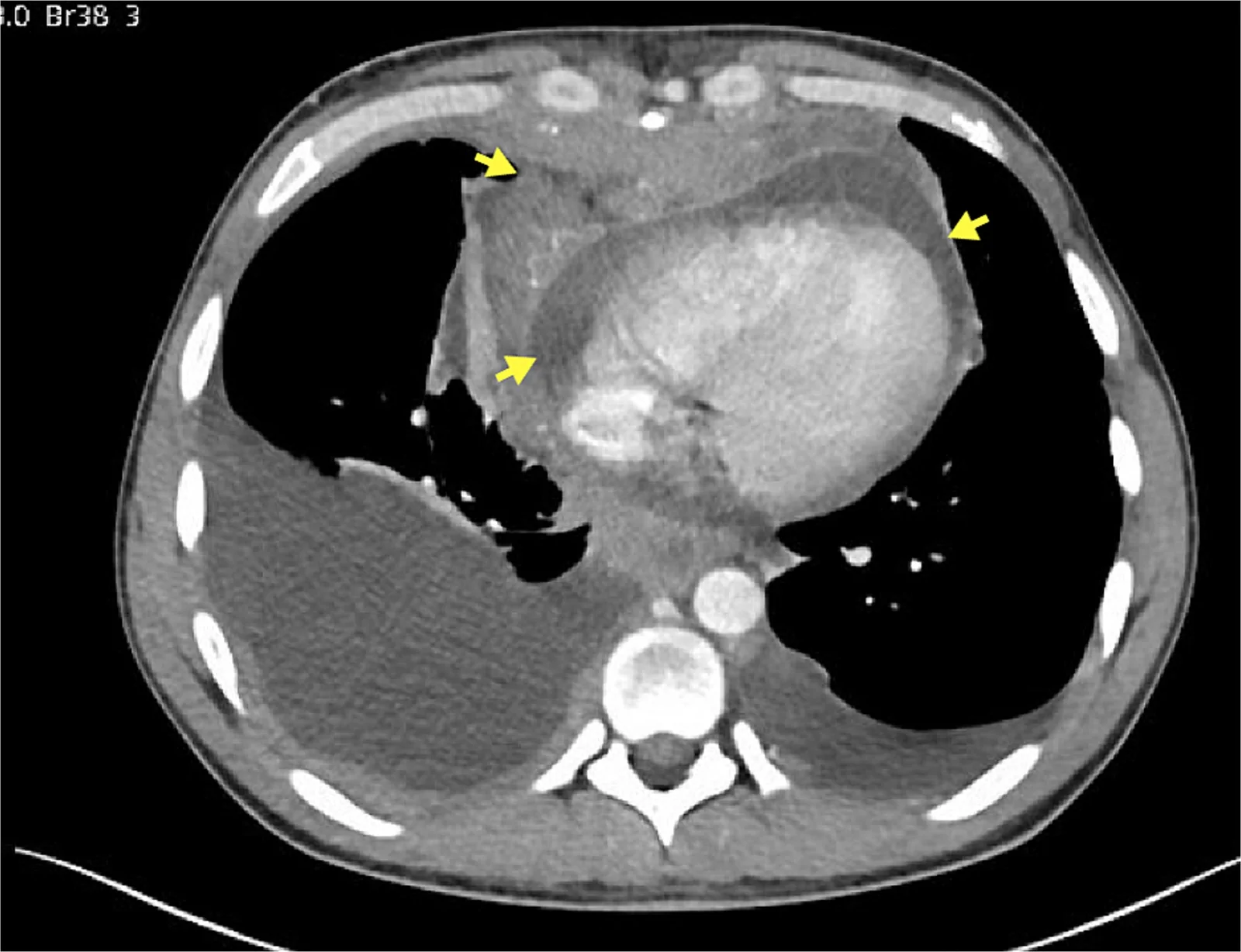
CT scan showing a large anterior mediastinal mass and pericardial effusion.

A CT-guided biopsy of the mediastinal mass was done, Histopathological analysis of the CT-guided mediastinal mass biopsy showed sheets of medium-sized lymphoid cells effacing the normal tissue architecture (Fig. 4A). Immunohistochemistry revealed strong positivity for CD10 (Fig. 4B), TdT (Fig. 4C), and CD3 (Fig. 4D), confirming the diagnosis of T-lymphoblastic leukemia/lymphoma (T-ALL/LBL). Whole-body Positron Emission Tomography (PET/CT) demonstrated mild-to-moderate Fluorodeoxyglucose (FDG) uptake in the mediastinal mass and associated lymph nodes in the supraclavicular, mediastinal, and upper retroperitoneal regions, with no evidence of axial skeleton or abdominal organ involvement (Fig. 5).

**Figure 4:**
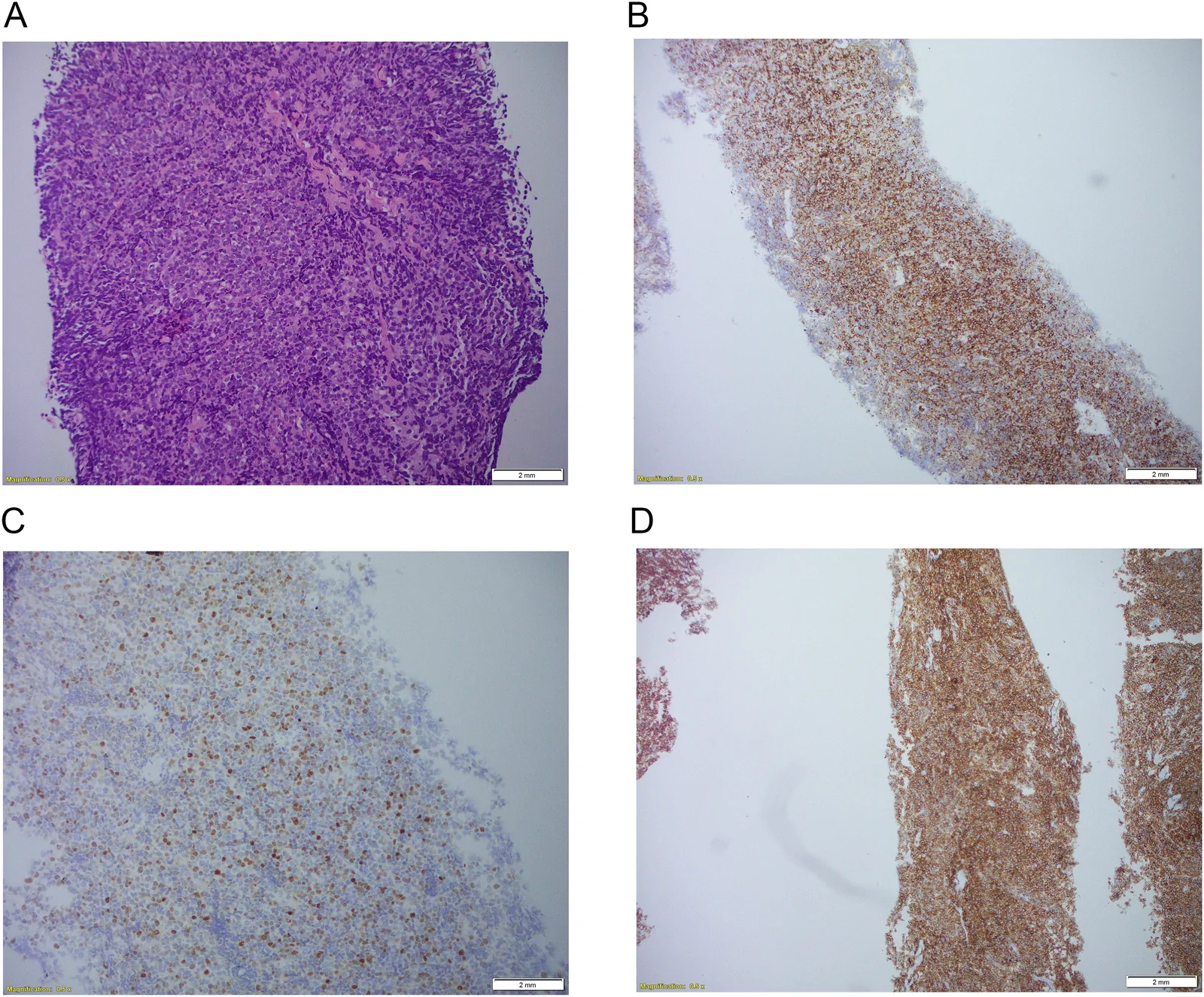
(A) H&E stain (×200) showing sheets of medium-sized lymphoid cells completely effacing the normal architecture. (B) Immunohistochemistry for CD10 showing strong positivity in neoplastic cells. (C) Immunohistochemistry for TdT showing strong positivity in neoplastic cells. (D) Immunohistochemistry for CD3 showing strong positivity in neoplastic cells.

**Figure 5:**
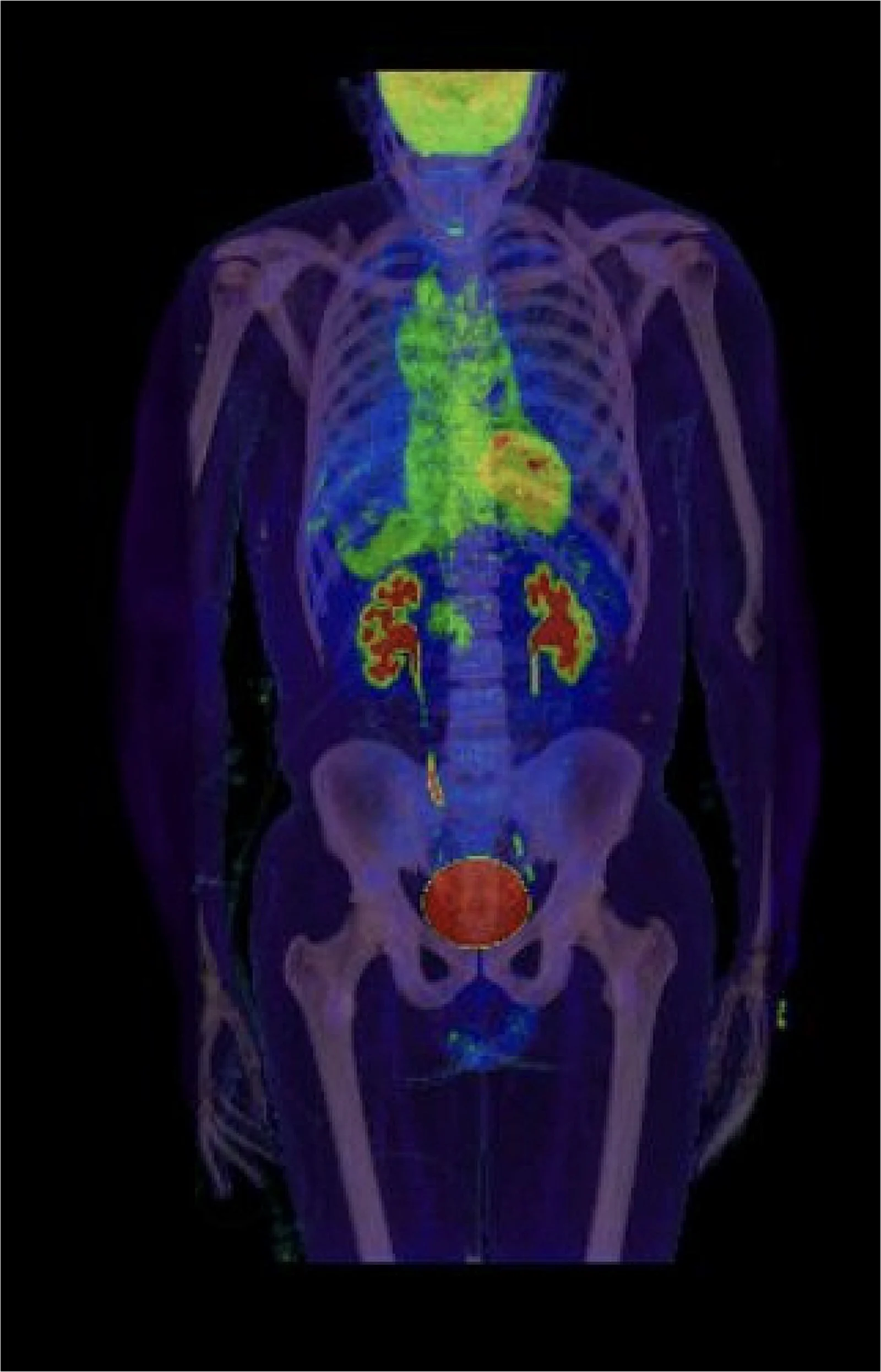
Whole-body PET/CT showed mild-to-moderate FDG uptake in the mediastinal mass.

The case was discussed in a multidisciplinary team (MDT) meeting, including the oncology team. Following a bone marrow aspiration that demonstrated normal trilineage hematopoiesis with no blast proliferation, the patient was initiated on the Berlin–Frankfurt–Münster (BFM) chemotherapy protocol, which is widely used for T-lymphoblastic leukemia/lymphoma [7].

The induction phase (Cycle 1A) was completed over 30 days without any treatment-related complications, followed by the early consolidation phase (Cycle 1B), completed over 45 days. The regimen included dexamethasone, daunorubicin, vincristine, and peg-asparaginase, consistent with the standard BFM protocol.

A post–Cycle 1B PET scan showed complete resolution of the previously noted infiltrative mediastinal mass and supraclavicular, mediastinal, and upper retroperitoneal lymphadenopathy. Pericardial effusions had fully resolved. Following the intensive treatment phase, the patient was transitioned to the maintenance phase and continues under regular hematology follow-up.

## Discussion

Malignant pericardial effusion is more commonly associated with metastatic disease rather than primary cardiac tumors. Notably, nearly 20% of unexplained pericardial effusions may represent an early manifestation of undiagnosed malignancy. T-ALL/LBL presented with cardiac tamponade is rare, with only a few cases documented in the literature. The presentation of pericardial tamponade is life-threatening and warrants immediate recognition and intervention [8]. The combination of signs known as Beck triad, namely low arterial blood pressure, dilated neck veins, and muffled heart sounds, are all present in only a minority of cases of acute cardiac tamponade [9]. Transthoracic echocardiography, in contrast, offers high diagnostic accuracy, allowing for the early detection of tamponade physiology and guiding the need for urgent pericardiocentesis, which in turn not only provides therapeutic relief, but can also help identify leukemic or lymphoid cells in the pericardial fluid.

In cases where patients present with pericardial effusion but no signs of tamponade, a thorough investigation is typically required before pericardiocentesis is performed. However, our patient exhibited classic tamponade physiology, necessitating immediate intervention. Pericardiocentesis and subsequent cytological analysis of the pericardial fluid were instrumental in establishing the diagnosis of T-ALL/LBL in a timely manner.

Secondary cardiac involvement in malignant lymphoma is generally a late manifestation of the disease. In contrast, our patient presented initially with cardiac tamponade as the primary manifestation [10]. In our case, we presume that the close anatomical proximity of the mediastinal mass to the heart facilitated direct extension of the tumor, increasing the likelihood of compression of cardiac chambers and obliteration of the pericardial space [11, 12].

A review of previously reported cases of T-ALL/LBL presenting with pericardial tamponade is summarized in Table 1. Most patients were young adults presenting with dyspnea and hemodynamic compromise; emergent pericardiocentesis followed by chemotherapy led to favorable outcomes in most cases.

**Table 1:** Reported cases of T-lymphoblastic Leukemia/lymphoma presenting as pericardial tamponade.

Author	Patient Age/Sex	Presentation	Key Findings	Intervention/Treatment	Outcome
Bacharou et al. [13]	17-year-old/Male	Dyspnea	Large pericardial effusion, right chamber collapse, non-compressible IVC; mediastinal mass 132 × 105 mm	Emergent pericardiocentesis, pleuropericardial biopsy; combination chemotherapy	Favorable response
Kapur & Levin [8]	25-year-old/Male	Progressive dyspnea, cough, hypotension, JVD, muffled heart sounds	Massive pericardial and pleural effusions, anterior mediastinal mass	Emergent pericardial and pleural drainage; histopathology + flow cytometry; chemotherapy	Favorable response
Cherala & Dao [14]	52-year-old/Female	Dyspnea, cough, back pain, night sweats	Mediastinal mass, pericardial effusion with tamponade physiology, hematologic abnormalities	Pericardiectomy, mass biopsy, hydroxyurea, hyper-CVAD, salvage chemo, matched sibling allogeneic stem cell transplant	Complete remission
Santos da Silva et al. [15]	24-year-old/Female	Progressive dyspnea	Large pericardial effusion with tamponade physiology; T-cell lymphoblastic lymphoma	Partial pericardiectomy, immunophenotyping; chemotherapy	Favorable response

Bacharou et al. reported a similar presentation in a 17-year-old adolescent with no significant medical history who presented with dyspnea. Echocardiography revealed a large pericardial effusion with right chamber collapse and non-compressible dilatation of the inferior vena cava. Emergent pericardiocentesis was performed, and thoracic CT showed a mediastinal mass measuring 132 × 105 mm. Pleuropericardial biopsy confirmed T-cell lymphoblastic lymphoma, and the patient was treated with combination chemotherapy [13].

Kapur and Levin reported a 25-year-old man presenting with progressive shortness of breath, cough, hypotension, jugular venous distention, muffled heart sounds, and massive pleural and pericardial effusions. Imaging and echocardiography revealed a large anterior mediastinal mass causing cardiac tamponade and bilateral pleural effusions. Emergent pericardial and pleural drainage was performed, and histopathology with flow cytometry confirmed precursor T-cell lymphoblastic lymphoma. He was subsequently treated with chemotherapy [8].

Cherala and Dao described a 52-year-old woman with a history of estrogen- and progesterone-positive, HER2-negative breast cancer who presented with dyspnea, cough, back pain, and night sweats. Workup revealed a mediastinal mass, pericardial effusion with tamponade physiology, and hematologic abnormalities. Pericardiectomy and mass biopsy confirmed acute T-cell lymphoblastic leukemia. She developed a pneumothorax and received hydroxyurea, hyper-CVAD chemotherapy, salvage chemotherapy, and a matched sibling allogeneic stem cell transplant, ultimately achieving complete remission [15].

Santos da Silva et al. reported a 24-year-old female presenting with progressive dyspnea due to a large pericardial effusion with tamponade physiology, which led to the diagnosis of T-cell lymphoblastic lymphoma. The patient underwent partial pericardiectomy, and immunophenotyping confirmed the diagnosis. Chemotherapy was initiated with a favorable response [15].

## Conclusion

While cardiac tamponade as an initial presentation of T-ALL or T-LBL is rare, this case highlights the importance of considering hematologic malignancies in the differential diagnosis of unexplained pericardial effusions. Early recognition and intervention are critical, as delays in diagnosis can result in significant morbidity and mortality.

## Data Availability

Not applicable.
